# A Systematic Review and Meta-Analysis of Ilizarov Methods in the Treatment of Infected Nonunion of Tibia and Femur

**DOI:** 10.1371/journal.pone.0141973

**Published:** 2015-11-03

**Authors:** Peng Yin, Qiunan Ji, Tongtong Li, Jiantao Li, Zhirui Li, Jianheng Liu, Guoqi Wang, Song Wang, Lihai Zhang, Zhi Mao, Peifu Tang

**Affiliations:** 1 Department of Orthopaedics, Chinese PLA General Hospital, No. 28 Fuxin Road, Beijing, 100853, P.R. China; 2 School of Medicine, Nankai University, No. 94 Weijin Road, Tianjin, 300071, P.R. China; 3 School of Medicine, The Chinese University of Hong Kong, Shatin, Hong Kong, China; Van Andel Institute, UNITED STATES

## Abstract

**Background:**

Infected nonunion of tibia and femur are common in clinical practice, however, the treatment of these diseases has still been a challenge for orthopaedic surgeons. Ilizarov methods can eradicate infection, compensate bone defects and promote the bone union through progressive bone histogenesis. The objective of this systematic review was to review current available studies reporting on Ilizarov methods in the treatment of infected nonunion of tibia and femur, and to perform meta-analysis of bone and functional results and complications to evaluate the efficacy of Ilizarov methods.

**Methods:**

A comprehensive literature search was performed from the SCI, PubMed, Cochrane Library; and Embase between January 1995 and August 2015. Some major data were statistically analyzed using weighted means based on the sample size in each study by SPSS 13.0, including number of patients, mean age, mean previous surgical procedures, mean bone defects, mean length of follow-up, bone union, complications per patient, external fixation time, and external fixation index(EFI). Bone results (excellent, good, fair and poor rate), functional results (excellent, good, fair and poor rate) and complications were analyzed by Stata 9.0.

**Findings:**

A total of 590 patients from 24 studies were included in this systematic review. The average of bone union rate was 97.26% in all included studies. The poor rate in bone results and functional results was 8% (95%CI, 0.04–0.12; I^2^ = 44.1%, P = 0.065) and 10% (95%CI, 0.05–0.14; I^2^ = 34.7%, P = 0.121) in patients with infected nonunion of tibia and femur treated by Ilizarov methods. The rate of refracture, malunion, infectious recurrence, knee stiffness, amputation, limb edema and peroneal nerve palsy was respectively 4%, 7%, 5%, 12%, 4%, 13% and 13%.

**Conclusions:**

Our systematic review showed that the patients with infected nonunion of tibia and femur treated by Ilizarov methods had a low rate of poor bone and functional results. Therefore, Ilizarov methods may be a good choice for the treatment of infected nonunion of tibia and femur.

## Introduction

Infected nonunion of tibia and femur are common in clinical practice [1], however, the treatment of these diseases has still been a challenge for orthopaedic surgeons **[** 2–5]. Some associated factors usually complicate the infected nonunion including bone and soft tissue loss, several sinuses, deformities, limb-length inequalities and polybacterial infection [6]. Several methods have been applied successfully in the treatment of infected nonunion of tibia and femur, including bone grafting, free tissue transfer and antibiotic cement, but these treatments have obvious limitations, such as donor site morbidity, stress fracture, and restriction of the size of bone defects[1]. Moreover, none of these treatments can afford surgeon the ability to treat infected nonunion associated with the mentioned factors simultaneously. The ability is possible with the application of Ilizarov methods. Ilizarov methods can eradicate infection, compensate bone defects and promote the bone union through progressive bone histogenesis [7], at the same time, it can correct the deformities and limb-length discrepancy during the course of bone transport[8].

Ilizarov methods base on the principles of distraction osteogenesis. It entails a segmental bone transport in which corticotomy is performed in the metaphysis and the bone is gradually distracted. Application of Ilizarov methods in the treatment of an infected nonunion depends on the extent of infection, the type of infected nonunion and the condition of the soft tissues[9]. In order to eliminate infection, it is critical to perform radical resection of the necrotic bone and infected segments [1]. Then internal bone transport is used to reconstruct the residual segmental defect [10,11].

Up to now, there are numerous reports on the treatment of infected nonunion of tibia and femur by Ilizarov methods, and it has gradually been a main treatment for infected nonunion. Although infected nonunion treated by Ilizarov methods acquired a satisfactory outcome in most studies, there were still some relative dissatisfactory results in several studies [7,12]. In addition, a relative high rate of complication by Ilizarov methods has been reported in some clinical researches [13–15]. However, no systematic review has been done to evaluate the effect of the treatment of infected nonunion of tibia and femur by Ilizarov methods. Therefore, we did a systematic review and meta-analysis of the scientific literature to evaluate and quantitate this effect, and try our best to give a valuable conclusion

## Materials and Methods

### Search Strategy

We did serial literature searches for relevant studies according to the guidelines from the Cochrane Collaboration. The following databases were searched: SCI (January 1995 to August 2015), PubMed (January 1995 to August 2015); Cochrane Library (January 1995 to August 2015); and Embase (January 1995 to August 2015).Keywords used to identify relevant articles were ‘infected’ or ‘infection’, ‘nonunion’, ‘non-union’, ‘tibia’, ‘femur’, ‘Ilizarov method’ or ‘Ilizarov methods’, and ‘Ilizarov technique’ or ‘Ilizarov techniques’. We used MeSH terms including ‘infection’, ‘tibia’, ‘femur’, and ‘Ilizarov technique’.

### Eligibility Criteria

The following eligibility criteria were performed in articles selection: (1) target population: patients with infected nonunion of tibia and femur; (2) intervention: Ilizarov methods, including bone transport, acute compression and lengthening, and compression osteosynthesis; (3) outcomes: bone union, bone results evaluated by ASAMI(rated as excellent, good, fair and poor), functional results evaluated by ASAMI(rated as excellent, good, fair and poor), complications, external fixation time and external fixation index. The eligible study included two above-mentioned outcomes at least; (4) article types: any type of the articles, excluding case report and review; (5) language restriction: articles written in the English language. We did the language restriction in order to avoid translation costs. Duplicate or multiple publications of the same study were excluded. We also excluded studies involving animal models, children, basic research, and when it was impossible to extract or calculate the data of infected nonunion from the studies.

### Data Extraction

All relevant data that met the eligibility criteria were independently and separately extracted by two authors. Discrepancies were resolved by discussion with each other. The following data were extracted from each included study: first author, publication year, study design, technique, site of infected nonunion, number of patients, mean age, mean previous surgical procedures, mean bone defects, mean length of follow-up, bone union, bone results evaluated by ASAMI, functional results evaluated by ASAMI, complications per patient, external fixation time, and external fixation index(EFI), complications (pin-track infection, axial deviation, bone grafting, loosening of wires, breakage of wires, malunion, refracture, knee stiffness, ankle stiffness, amputation, limb edema and peroneal nerve palsy).

### Data Analysis

Bone results (excellent, good, fair and poor rate), functional results (excellent, good, fair and poor rate) and complications were analyzed by using STATA 9.0. Differences were expressed as effect size (ES) with 95% CIs for the rate meta-analysis. Heterogeneity among studies was tested by using the standard chi-square test(with significance defined as P<0.1), and the I-square test (with a value greater than 50% representing substantial heterogeneity)[16]. A random effect model was chosen regardless of heterogeneity. Because the sites of infected nonunion were inconsistent among studies, we further conducted subgroup analyses to explore possible explanations for heterogeneity and examine the influence of various overall pooled estimate. We also tested the influence of a single study on the overall pooled estimate by omitting one study in each turn, if the study reported bone results and/or functional results. Other major data extracted in this study were recorded and statistically analyzed using weighted means based on the sample size in each study by SPSS 13.0, including number of patients, mean age, mean previous surgical procedures, mean bone defects, mean length of follow-up, bone union, complications per patient, external fixation time, and external fixation index(EFI). The remaining data was analyzed by description from original studies.

## Results

### Literature Search

**Fig 1:**
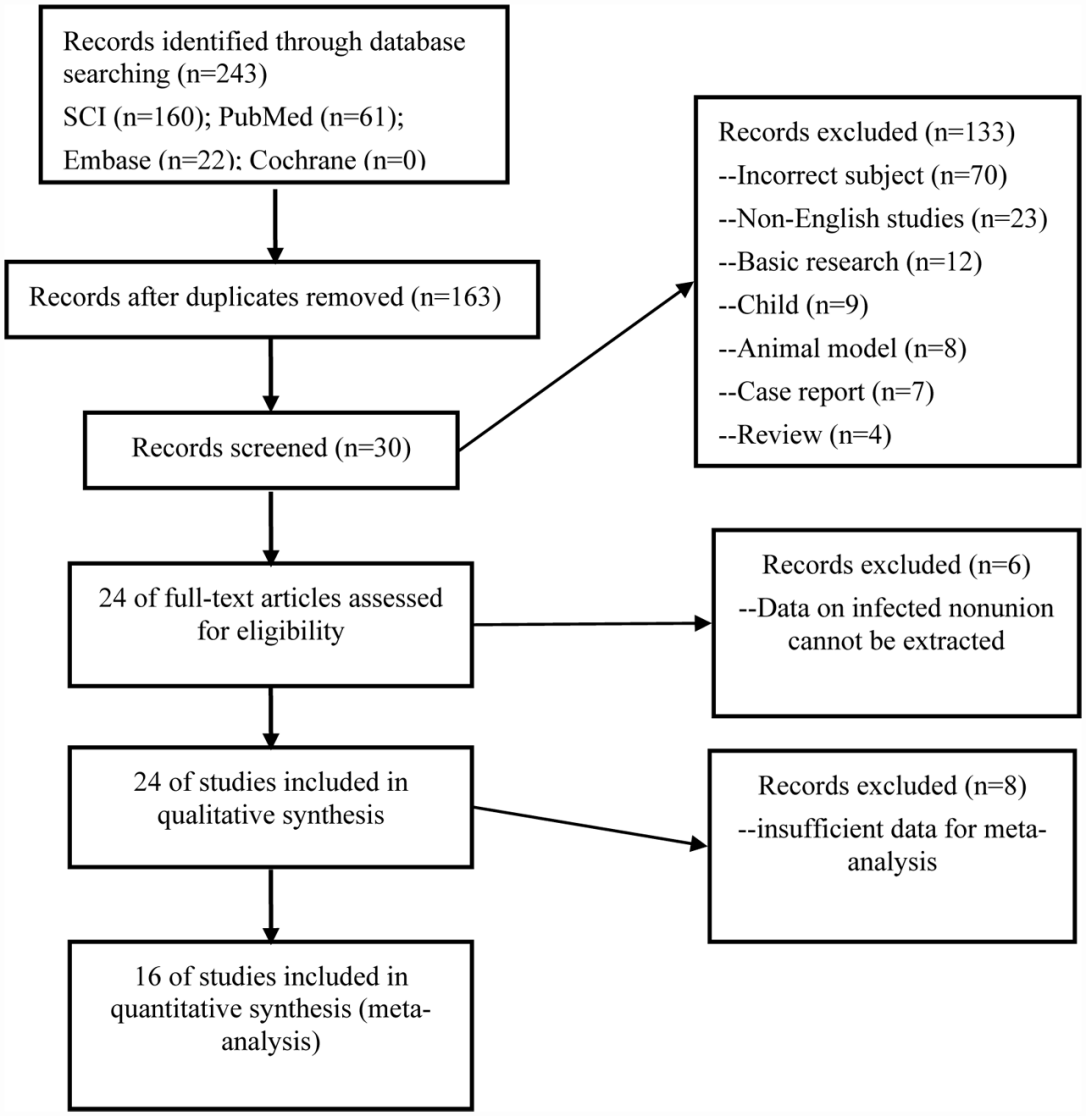
Flow chart illustrating number of studies evaluated at each stage in the systematic review.

The initial literature search identified 243 relevant records published from January 1995 to August 2015. 30 studies remained after screening by reading titles and abstracts. Ultimately, 24 studies met the inclusion and exclusion criteria in the systematic review by reviewing the full-text articles(Fig 1)[1,7,8,10–15,17–31].Of the included studies, 22 were retrospective case series[1,8,10–15,17–21,23–31],1 was retrospective comparative study[22], and 1 was prospective comparative study[7].

### Patient Information

The systematic review included a total of 590 patients with infected nonunion of tibia and femur treated by Ilizarov methods. The mean age of all patients was 33.79 years; the mean age was 34.11 years in patients with infected tibia nonunion and 32.68 years in patients with infected femur nonunion. The patients had an average of 3.64 previous surgical procedures before receiving the treatment of Ilizarov method[1,7,8,10,12–15,17–21,23,24,27,29–31]; the mean previous surgical procedures was 3.84 in patients with infected tibia nonunion and 3.81 in patients with infected femur nonunion. The mean bone defects was 6.70 cm in the patients[1,7,8,10–15,17–25,27–31], and 6.54 cm in patients with infected tibia nonunion and 8.05 cm in patients with infected femur nonunion. The mean length of follow-up was 39.79 months in the patients[1,7,8,10–14,17–24,26–31], and 32.49 months in patients with infected tibia nonunion and 64.47 months in patients with infected femur nonunion. Further details were listed in Table 1.

**Table 1:** Characteristics of included studies.

Author	Study No.	Year	Study design	Number of patients	Mean age (years)	MPSP (per patient)	Mean bone defects(cm)	Follow-up (months)
Yin^1^	1	2015	RS	72	38.45	2.55	6.46	24.13
Khan^10^	2	2015	RS	24	38	2	3.3	11
Peng^17^	3	2015	RS	58	29.4	6.3	9.2	31.6
Xu^18^	4	2014	RS	30	34.1	6	6.43	29
Feng^19^	5	2013	RS	21	34.6	6	6.6	31
Blum^13^	6	2010	RS	50	29.9	3.8	8.8	70.8
Megas^20^	7	2010	RS	9	39.7	4.8	5	26.6
Bumbasirevic^4^	8	2010	RS	30	30.4	1.3	6.9	99
Emara^22^	9	2008	RC	33	29	__	6	36
Madhusudhan^7^	10	2008	PC	22	37.2	3	4/5.4*	13
Rose^12^	11	2007	RS	6	31.83	3.83	4.33	7.6
Magadum^24^	12	2006	RS	27	39	2	10	27
Krishnan^14^	13	2006	RS	20	38.4	4.4	6	63
Saridis^23^	14	2006	RS	13	34.6	3	8.3	42.4
Abdel-Aal^25^	15	2006	RS	9	30.66	__	10.7	__
McHale^26^	16	2004	RS	10	31	__	__	36
Arora^27^	17	2003	RS	46	35	2.1	6	67
Atesalp^28^	18	2002	RS	14	25	__	4.4	33.2
Barbarossa^15^	19	2001	RS	23	40.7	4.2	6.2	__
Maini^29^	20	2000	RS	15	27.4	2.5	7	31.2
Laursen^30^	21	2000	RS	9	25.78	6.9	4.89	39.4
Ring^11^	22	1999	RS	10	34	__	4.3	7.2
Hosny^8^	23	1998	RS	11	27	2	3.7	13
Dendrinos^31^	24	1995	RS	28	37	4	6	39
Total number of patients				590				

MPSP mean previous surgical procedures RS retrospective case series RC retrospective comparative study

PC prospective comparative study

__ The data did not be reported in studies.

* The study included two groups, the mean bone defects is 4cm in one group, and 5.4cm in another group.

### Interventions and Outcomes

The interventions mainly included three parts: radical debridement, antibiotic treatment, and Ilizarov methods. Ilizarov methods included three techniques: bone transport, acute compression and lengthening, and compression osteosynthesis. Flap transfer was reported in 2 included studies [11,28]. Bone grafting as a routine treatment was recommended in 1 included study[22].

The average of bone union rate was 97.26% in all included studies, and 97.50% in the studies of infected tibia nonunion and 97.59% in the studies of infected femur nonunion. The mean complications of every patient were 1.36 in all patients, and 1.23 in patients with infected tibia nonunion and 2.24 in patients with infected femur nonunion. The mean external fixation time was 10.69 months in the patients[1,7,8,10–14,17–28,30,31], and 9.41 months in patients with infected tibia nonunion and 18.26 months in patients with infected femur nonunion. The mean external fixation index was 1.70 months/cm in the patients[1,7,8,10,13,14,17–24,26–28,31], and 1.64 months/cm in patients with infected tibia nonunion and 2.19 months/cm in patients with infected femur nonunion. Further details were listed in Table 2.

**Table 2:** Interventions and Outcomes of included studies.

Study No.	Technique	Site	Bone union No.(%)	Bone results(ASAMI) (excellent/good/fair/poor)	Functional results(ASAMI) (excellent/good/fair/poor)	Complications (per patient)	EFT (months)	EFI (Ms/cm)
1	RD,AT,BT(IEF)	72T	72/72(100%)	46/17/7/2	25/27/13/0*	1.10(79/72)	9.56	1.48
2	RD,AT,BT or CO (IEF)	24T	22/23(95.7%)#	6/14/1/2	8/12/2/0(1 failure)	0.5(12/24)	8	4.2
3	RD,AT,BT(IEF)	58T	58/58(100%)	30/23/5/0	28/18/12/0	0.67(39/58)	10.6	1.2
4	RD,AT,BT(IEF)	30 T	30/30(100%)	28/2/0/0	__	0.27(8/30)	10	1.37
5	RD,AT,BT(IEF)	21 T	21/21(100%)	19/2/0/0	__	0.4(8/21)	9.8	1.48
6	RD,AT,BT(IEF)	50 F	49/50(98%)	__	__	2.1(105/50)	24.5	2.8
7	RD,AT,CO or ACL(IEF)	9 T	9/9(100%)	5/4/0/0	3/4/2/0	1.89(17/9)	7.83	1.07
8	RD,AT,BT(IEF)	30 T	29/30(97%)	19/10/0/1	13/14/2/1	1.4(42/30)	9.7	1.48
9	RD,AT, BT(IEF),BG	16 T	16/16(100%)	15/1/0/0	12/1/3/0	0.4(6/16)	8.5	1.5
	RD,AT, BT(IEF and IMN),BG	17T	17/17(100%)	17/0/0/0	13/2/2/0	0.12(2/17)	3.1	0.55
10	RD, AT,ACL(IEF)	13 T	13/13(100%)	4/3/4/2	1/3/6/2*	2.73(60/22)**	9.3	2.33
	RD, AT,BT(IEF)	9 T	9/9(100%)	0/3/4/2	0/1/3/2***		8.5	1.57
11	RD,AT,CO or BT(IEF)	5 T/1 F	5/6(83.3%)	1/3/1/1	1/3/0/2	1.33(8/6)	10	__
				(5T+1F)	(5T+1F)			
12	RD, ACL(IEF)	27 T	24/25(96%)****	19/5/0/1	15/8/1/1	1.16(29/25)	10.2	1.02
13	RD,AT,BT or ACL(IEF)	20 F	19/20(95%)	13/4/1/1(1AMP)	3/9/3/4(1AMP)	3.55(71/20)	7.8	1.28
14	RD,AT,ACL or BT(IEF)	13 F	13/13(100%)	8/4/1/0	3/4/4/2	0.76(10/13)	10.33	1.24
15	RD,BT(IEF)	9 T	9/9(100%)	__	__	1.11(10/9)	12.78	1.22
16	RD,AT,BT or ACL or CO(IEF)	10 T	10/10(100%)	__	__	0.4(4/10)	9.0	__
17	RD,BT or CO(IEF)	38 T/8 F	44/46(95.4%)	__	15/16/13/2	0.74(34/46)	8.7	1.33
18	RD,AT,3 flaps, BT(IEF)	14 T	13/14(92.9%)	__	__	1.21(17/14)	6.8	1.55
19	RD,AT,BT(IEF)	23 T	20/23(87%)	8/8/2/4(1AMP)	2/10/6/4(1AMP)	3.39(78/23)	__	__
20	RD,AT,BT(IEF)	3 F/12 T	15/15(100%)	7/3/0/5	4/7/1/3	2.27(34/15)	__	__
21	RD,AT,CO or BT(IEF)	9 T	9/9(100%)	__	__	1.56(14/9)	6.7	__
22	RD,3flaps,BT or ACL or CO(IEF)	10 T	9/10(90%)	__	__	2.5(25/10)	6.9	__
23	RD,3AT, BT or CO(IEF)	11 T	11/11(100%)	__	5/3/2/1	1.27(14/11)	8.5	2.3
24	RD,BT(IEF)	28 T	25/28(89%)	14/8/1/5	7/11/4/5(1AMP)	2.5(71/28)	10	1.67

^#^ 1 patient die for advanced liver disease

* 7 patient lost for follow up

**3 patients were unable to evaluate

***Complications did not be recorded separately by groups

****2 patients lost for follow up

__ The data did not be reported in studies.

ACL acute compression and lengthening AMP amputation ASAMI Association for the Study of the Method of Ilizarov AT antibiotics treatment BG bone graft BT bone transport CO compression osteosynthesis EFI external fixation index EFT external fixation time F femur IEF Ilizarov external fixator IMN intramedullary nailing Ms/cm months/cm RD radical debridement T tibia

### Bone Results and Functional Results

The criteria recommended by ASAMI were adopted to evaluate bone results and functional results in the studies[1,7,8,10,12,14,15,17–24,27,29,31]. Bone results were evaluated by 4 criteria: union, infection, deformity and limb-length discrepancy. Functional results were evaluated by 5 criteria: active, limp, minimum stiffness (knee or ankle joint), reflex sympathetic dystrophy and pain.

Bone results were evaluated in 16 studies by ASAMI [1,7,10,12,14,15,17–24,29,31]. Random effects meta-analysis showed that the weighted frequency of excellent rate, good rate, fair rate and poor rate in bone results were listed in Table 3.

**Table 3:** Meta-analysis of bone results and functional results evaluated by ASAMI.

Results	Relevant studies (n)	Heterogeneity(I^2^,%; P)	ES(95% CI)	Range of incidence (%)
**Bone results**				
Rate of excellent results	16^[^ ^1^ ^,^ ^7^ ^,^ ^10^ ^,^ ^12^ ^,^ ^14^ ^,^ ^15^ ^,^ ^17^ ^–^ ^24^ ^,^ ^29^ ^,^ ^31^ ^]^	I^2^ = 93.1; P = 0.000	0.58 (0.44,0.72)	17–97
Rate of good results	16^[^ ^1^ ^,^ ^7^ ^,^ ^10^ ^,^ ^12^ ^,^ ^14^ ^,^ ^15^ ^,^ ^17^ ^–^ ^24^ ^,^ ^29^ ^,^ ^31^ ^]^	I^2^ = 80.8; P = 0.000	0.26 (0.18,0.34)	3–61
Rate of fair results	9^[^ ^1^ ^,^ ^7^ ^,^ ^10^ ^,^ ^12^ ^,^ ^14^ ^,^ ^15^ ^,^ ^17^ ^,^ ^23^ ^,^ ^31^ ^]^	I^2^ = 26.9; P = 0.205	0.08 (0.04,0.12)	4–36
Rate of poor results	10^[^ ^1^ ^,^ ^7^ ^,^ ^10^ ^,^ ^12^ ^,^ ^14^ ^,^ ^15^ ^,^ ^21^ ^,^ ^24^ ^,^ ^29^ ^,^ ^31^ ^]^	I^2^ = 44.1; P = 0.065	0.08 (0.04,0.12)	3–33
**Functional results**				
Rate of excellent results	16^[^ ^1^ ^,^ ^7^ ^,^ ^8^ ^,^ ^10^ ^,^ ^12^ ^,^ ^14^ ^,^ ^15^ ^,^ ^17^ ^,^ ^20^ ^–^ ^24^ ^,^ ^27^ ^,^ ^29^ ^,^ ^31^ ^]^	I^2^ = 84.8; P = 0.000	0.33 (0.23,0.44)	6–76
Rate of good results	16^[^ ^1^ ^,^ ^7^ ^,^ ^8^ ^,^ ^10^ ^,^ ^12^ ^,^ ^14^ ^,^ ^15^ ^,^ ^17^ ^,^ ^20^ ^–^ ^24^ ^,^ ^27^ ^,^ ^29^ ^,^ ^31^ ^]^	I^2^ = 59.3; P = 0.001	0.36 (0.28,0.43)	9–52
Rate of fair results	15^[^ ^1^ ^,^ ^7^ ^,^ ^8^ ^,^ ^10^ ^,^ ^14^ ^,^ ^15^ ^,^ ^17^ ^,^ ^20^ ^–^ ^24^ ^,^ ^27^ ^,^ ^29^ ^,^ ^31^ ^]^	I^2^ = 56.4; P = 0.004	0.17 (0.11,0.22)	4–50
Rate of poor results	11^[^ ^7^ ^,^ ^8^ ^,^ ^12^ ^,^ ^14^ ^,^ ^15^ ^,^ ^21^ ^,^ ^23^ ^,^ ^24^ ^,^ ^27^ ^,^ ^29^ ^,^ ^31^ ^]^	I^2^ = 34.7; P = 0.121	0.10 (0.05,0.14)	3–33

Functional results were reported in 16 studies [1,7,8,10,12,14,15,17,20–24,27,29,31]. Random effects meta-analysis showed that the weighted frequency of excellent rate, good rate, fair rate and poor rate in functional results were listed in Table 3.

Table 4 showed subgroup analysis of bone results and functional results evaluated by ASAMI based on the sites of infected nonunion.

**Table 4:** Subgroup analysis of bone results and functional results evaluated by ASAMI based on the sites of infected nonunion.

Results	Relevant studies (n)	Heterogeneity(I^2^,%; P)	ES(95% CI)	Range of incidence (%)
**Tibia**				
**Bone results**				
Rate of excellent results	12^[^ ^1^ ^,^ ^7^ ^,^ ^10^ ^,^ ^15^ ^,^ ^17^ ^–^ ^22^ ^,^ ^24^ ^,^ ^31^ ^]^	I^2^ = 94.4; P = 0.000	0.61 (0.45,0.77)	18–97
Rate of good results	12^[^ ^1^ ^,^ ^7^ ^,^ ^10^ ^,^ ^15^ ^,^ ^17^ ^–^ ^22^ ^,^ ^24^ ^,^ ^31^ ^]^	I^2^ = 85.2; P = 0.000	0.26 (0.16,0.36)	3–61
Rate of fair results	6^[^ ^1^ ^,^ ^7^ ^,^ ^10^ ^,^ ^15^ ^,^ ^17^ ^,^ ^31^ ^]^	I^2^ = 51.5; P = 0.067	0.09 (0.03,0.14)	4–36
Rate of poor results	7^[^ ^1^ ^,^ ^7^ ^,^ ^10^ ^,^ ^15^ ^,^ ^21^ ^,^ ^24^ ^,^ ^31^ ^]^	I^2^ = 40.8; P = 0.119	0.07 (0.02,0.11)	3–18
**Functional results**				
Rate of excellent results	11^[^ ^1^ ^,^ ^7^ ^,^ ^8^ ^,^ ^10^ ^,^ ^15^ ^,^ ^17^ ^,^ ^20^ ^–^ ^22^ ^,^ ^24^ ^,^ ^31^ ^]^	I^2^ = 89.2; P = 0.000	0.38 (0.23,0.52)	6–76
Rate of good results	11^[^ ^1^ ^,^ ^7^ ^,^ ^8^ ^,^ ^10^ ^,^ ^15^ ^,^ ^17^ ^,^ ^20^ ^–^ ^22^ ^,^ ^24^ ^,^ ^31^ ^]^	I^2^ = 69.7; P = 0.000	0.34 (0.25, 0.44)	9–52
Rate of fair results	11^[^ ^1^ ^,^ ^7^ ^,^ ^8^ ^,^ ^10^ ^,^ ^15^ ^,^ ^17^ ^,^ ^20^ ^–^ ^22^ ^,^ ^24^ ^,^ ^31^ ^]^	I^2^ = 59.1; P = 0.007	0.16 (0.10,0.22)	4–50
Rate of poor results	6^[^ ^7^ ^,^ ^8^ ^,^ ^15^ ^,^ ^21^ ^,^ ^24^ ^,^ ^31^ ^]^	I^2^ = 40.0; P = 0.139	0.09 (0.03,0.15)	3–22
**Femur**				
**Bone results**				
Rate of excellent results	2^[^ ^14^ ^,^ ^23^ ^]^	I^2^ = 0; P = 0.839	0.64 (0.47,0.80)	61–65
Rate of good results	2^[^ ^14^ ^,^ ^23^ ^]^	I^2^ = 0; P = 0.489	0.24 (0.09,0.38)	20–31
Rate of fair results	2^[^ ^14^ ^,^ ^23^ ^]^	I^2^ = 0; P = 0.760	0.06 (-0.02,0.14)	5–8
Rate of poor results	1^[^ ^14^ ^]^	_	0.05 (-0.05,0.15)	5
**Functional results**				
Rate of excellent results	2^[^ ^14^ ^,^ ^23^ ^]^	I^2^ = 0; P = 0.567	0.18 (0.05,0.30)	15–23
Rate of good results	2^[^ ^14^ ^,^ ^23^ ^]^	I^2^ = 0; P = 0.402	0.39 (0.22,0.55)	31–45
Rate of fair results	2^[^ ^14^ ^,^ ^23^ ^]^	I^2^ = 8.8; P = 0.295	0.20 (0.06,0.34)	15–31
Rate of poor results	2^[^ ^14^ ^,^ ^23^ ^]^	I^2^ = 0; P = 0.732	0.18 (0.05,0.31)	15–20

### Complications

Complications were summarized in Table 5. Subgroup analysis of complications based on the sites of infected nonunion was performed and the outcomes were listed in Table 6.

**Table 5:** Meta-analysis of complications of infected nonunion of tibia and femur treated by Ilizarov methods.

Complications	Relevant studies (n)	Heterogeneity(I^2^,%; P)	ES(95% CI)	Range of incidence (%)
Pin-track infection	23^[^ ^1^ ^,^ ^7^ ^,^ ^8^ ^,^ ^10^ ^–^ ^15^ ^,^ ^17^ ^–^ ^25^ ^,^ ^27^ ^–^ ^31^ ^]^	I^2^ = 97.6; P = 0.000	0.56 (0.43,0.69)	10–100
Axial deviation	6^[^ ^1^ ^,^ ^14^ ^,^ ^15^ ^,^ ^20^ ^,^ ^29^ ^,^ ^31^ ^]^	I^2^ = 76.5; P = 0.001	0.40 (0.25,0.56)	22–70
Bone grafting	5^[^ ^1^ ^,^ ^11^ ^,^ ^13^ ^,^ ^25^ ^,^ ^29^ ^]^	I^2^ = 56.4; P = 0.057	0.20 (0.09,0.31)	10–30
Loosening of wires	9^[^ ^1^ ^,^ ^7^ ^,^ ^8^ ^,^ ^11^ ^–^ ^13^ ^,^ ^15^ ^,^ ^22^ ^,^ ^27^ ^]^	I^2^ = 64.7; P = 0.004	0.15 (0.08,0.22)	6–48
Breakage of wires	5^[^ ^7^ ^,^ ^17^ ^–^ ^19^ ^,^ ^27^ ^]^	I^2^ = 57.1; P = 0.054	0.05 (0.00,0.09)	2–32
Knee stiffness	4^[^ ^8^ ^,^ ^11^ ^,^ ^17^ ^,^ ^29^ ^]^	I^2^ = 1.6; P = 0.384	0.12 (0.05,0.19)	9–30
Ankle stiffness	4^[^ ^11^ ^,^ ^20^ ^,^ ^22^ ^,^ ^29^ ^]^	I^2^ = 64.9; P = 0.036	0.31 (0.11,0.52)	13–56
Malunion	8^[^ ^10^ ^,^ ^11^ ^,^ ^15^ ^,^ ^18^ ^–^ ^20^ ^,^ ^24^ ^,^ ^26^ ^]^	I^2^ = 0; P = 0.570	0.07 (0.03,0.11)	4–22
Refracture	9^[^ ^1^ ^,^ ^7^ ^,^ ^10^ ^,^ ^14^ ^,^ ^15^ ^,^ ^23^ ^,^ ^28^ ^–^ ^30^ ^]^	I^2^ = 0; P = 0.931	0.04 (0.02,0.07)	3–13
Infectious recurrence	7^[^ ^10^ ^,^ ^11^ ^,^ ^17^ ^,^ ^22^ ^,^ ^26^ ^,^ ^28^ ^,^ ^29^ ^]^	I^2^ = 24.2; P = 0.245	0.05 (0.01,0.10)	2–30
Limb edema	3^[^ ^8^ ^,^ ^15^ ^,^ ^31^ ^]^	I^2^ = 0; P = 0.890	0.13 (0.04,0.21)	9–14
Amputation	4^[^ ^11^ ^,^ ^14^ ^,^ ^15^ ^,^ ^31^ ^]^	I^2^ = 0; P = 0.936	0.04 (0.00,0.09)	4–10
Peroneal nerve palsy	2^[^ ^8^ ^,^ ^11^ ^]^	I^2^ = 0; P = 0.585	0.13 (-0.01,0.28)	10–18

**Table 6:** Subgroup analysis of complications based on the sites of infected nonunion.

Complications	Relevant studies (n)	Heterogeneity(I^2^,%; P)	ES(95% CI)	Range of incidence (%)
**Tibia**				
Pin-track infection	17^[^ ^1^ ^,^ ^7^ ^,^ ^8^ ^,^ ^10^ ^,^ ^11^ ^,^ ^15^ ^,^ ^17^ ^–^ ^22^ ^,^ ^24^ ^,^ ^25^ ^,^ ^28^ ^,^ ^30^ ^,^ ^31^ ^]^	I^2^ = 97.2; P = 0.000	0.48 (0.29,0.68)	10–100
Axial deviation	4^[^ ^1^ ^,^ ^15^ ^,^ ^20^ ^,^ ^31^ ^]^	I^2^ = 62.3; P = 0.047	0.38 (0.23,0.53)	22–52
Bone grafting	3^[^ ^1^ ^,^ ^11^ ^,^ ^25^ ^]^	I^2^ = 19.6; P = 0.288	0.14 (0.03,0.24)	10–30
Loosening of wires	6^[^ ^1^ ^,^ ^7^ ^,^ ^8^ ^,^ ^11^ ^,^ ^15^ ^,^ ^22^ ^]^	I^2^ = 73.8; P = 0.002	0.17 (0.05,0.28)	6–48
Breakage of wires	4^[^ ^7^ ^,^ ^17^ ^–^ ^19^ ^]^	I^2^ = 67.2; P = 0.028	0.06 (-0.01,0.12)	2–32
Knee stiffness	3^[^ ^8^ ^,^ ^11^ ^,^ ^17^ ^]^	I^2^ = 15.5; P = 0.306	0.13 (0.03,0.22)	9–30
Ankle stiffness	3^[^ ^11^ ^,^ ^20^ ^,^ ^22^ ^]^	I^2^ = 62.8; P = 0.068	0.26 (0.04,0.49)	13–56
Malunion	8^[^ ^10^ ^,^ ^11^ ^,^ ^15^ ^,^ ^18^ ^–^ ^20^ ^,^ ^24^ ^,^ ^26^ ^]^	I^2^ = 0; P = 0.570	0.07 (0.03,0.11)	4–22
Refracture	6^[^ ^1^ ^,^ ^7^ ^,^ ^10^ ^,^ ^15^ ^,^ ^28^ ^,^ ^30^ ^]^	I^2^ = 0; P = 0.885	0.04 (0.01,0.07)	3–11
Infectious recurrence	6^[^ ^10^ ^,^ ^11^ ^,^ ^17^ ^,^ ^22^ ^,^ ^26^ ^,^ ^28^ ^]^	I^2^ = 34.5; P = 0.178	0.06 (0.00,0.11)	2–30
Limb edema	3^[^ ^8^ ^,^ ^15^ ^,^ ^31^ ^]^	I^2^ = 0; P = 0.890	0.13 (0.04,0.21)	9–14
Amputation	3^[^ ^11^ ^,^ ^15^ ^,^ ^31^ ^]^	I^2^ = 0; P = 0.817	0.04 (-0.01,0.09)	4–10
Peroneal nerve palsy	2[^8^ ^,^ ^11^ ^]^	I^2^ = 0; P = 0.585	0.13 (-0.01,0.28)	10–18
**Femur**				
Pin-track infection	3^[^ ^13^ ^,^ ^14^ ^,^ ^23^ ^]^	I^2^ = 90.3; P = 0.000	0.77 (0.45,1.09)	55–100
Axial deviation	1^[^ ^14^ ^]^	_	0.70 (0.50,0.90)	70
Bone grafting	1^[^ ^13^ ^]^	_	0.30 (0.17,0.43)	30
Loosening of wires	1^[^ ^13^ ^]^	_	0.08 (0.00,0.16)	8
Breakage of wires	1^[^ ^27^ ^]^	_	0.04 (-0.02,0.10)	4
Refracture	2^[^ ^14^ ^,^ ^23^ ^]^	I^2^ = 0; P = 0.761	0.06 (-0.02,0.14)	5–8
Amputation	1^[^ ^14^ ^]^	_	0.05 (-0.05,0.15)	5

## Discussion

This is the first systematic review of infected nonunion of tibia and femur treated by Ilizarov methods. The systematic review included 24 studies, and we conducted a meta-analysis of 16 studies to evaluate the efficacy of Ilizarov methods in the treatment of infected nonunion of tibia and femur. The poor rate in bone results and functional results was 8% (95%CI, 0.04–0.12; I^2^ = 44.1%, P = 0.065) and 10% (95%CI, 0.05–0.14; I^2^ = 34.7%, P = 0.121). The data were not statistically heterogeneous. Therefore, our results showed that the patients with infected nonunion of tibia and femur treated by Ilizarov methods had a low rate of poor bone and functional results.

We did a meta-analysis of complication in patients with infected nonunion of tibia and femur treated by Ilizarov method. Statistically homogeneity was found in most of the complications (Table 4). The rate of refracture and amputation was 4% and 4% in our study, which is similar with the 5% and 2.9% reported by Papakostidis et al[32]. The rate of peroneal nerve palsy was 13% in our study, which is higher than the 2.2% neurovascular complications reported by Papakostidis et al[32]. We considered that the reason was the different characteristics of included patients. The rate of malunion, infectious recurrence, limb edema, and knee stiffness was respectively 7%, 5%, 13% and 12%. The rate of infectious recurrence is lower than the rate in the study by Struijs using other treatments[33]. Pin-track infection is the most common complication by using Ilzarov methods, and significant statistically heterogeneity was found in the complication. The heterogeneity was still found after performing the subgroup analysis. The rate of pin-track infection was 10%-100% among included studies in our systematic review. Hence, we considered that meticulous pin care was the key to decreasing the complication.

In our systematic review, most studies involved infected tibia nonunion, and we performed subgroup analysis based on the sites of infected nonunion. The data of infected tibia nonunion could be found in Tables 4 and 6. The poor rate in bone results and functional results was 7% (95%CI, 0.02–0.11; I^2^ = 40.8%, P = 0.119) and 9% (95%CI, 0.03–0.15; I^2^ = 40.0%, P = 0.139). The rate of bone grafting, knee stiffness, malunion, refracture, infectious recurrence, limb edema, amputation and Peroneal nerve palsy was respectively 14%, 13%, 7%, 4%, 6%, 13%, 4% and 13%. These data were not statistically heterogeneous.

To our best knowledge, this is the first systematic review of infected nonunion of tibia and femur treated by Ilizarov methods. We were able to provide a large number of data on characteristics of patients and treatment results through 24 included studies. We also conducted meta-analyses of bone and functional results in our systematic review. High heterogeneity existed in several pooling data in our study, and we thought the heterogeneity was probably resulted from different research quality, various surgeons’ experience and diversity of rehabilitation nursing. Failure to include the non-English language studies in our article could have resulted in missing data and our estimates of effect size might have been biased, nevertheless, 24 studies were included in our article and they were not unduly affected by significant statistical heterogeneity. The data of the present review were extracted from observational studies, which are prone to cause both systematic and random error [34–37]. Therefore, more prospective randomized controlled trials are needed to overcome the limitation of our study.

In conclusion, our systematic review showed that the patients with infected nonunion of tibia and femur treated by Ilizarov methods had a low rate of poor bone and functional results. Therefore, Ilizarov methods may be a good choice for the treatment of infected nonunion of tibia and femur.
